# Development and validation of a diabetes risk score among two populations

**DOI:** 10.1371/journal.pone.0245716

**Published:** 2021-01-25

**Authors:** Natalie V. Schwatka, Derek E. Smith, Ashley Golden, Molly Tran, Lee S. Newman, Donna Cragle

**Affiliations:** 1 Center for Health, Work & Environment, Colorado School of Public Health, University of Colorado, Anschutz Medical Campus, Aurora, Colorado, United States of America; 2 Department of Environmental and Occupational Health, Colorado School of Public Health, University of Colorado, Anschutz Medical Campus, Aurora, Colorado, United States of America; 3 Department of Pediatrics, Cancer Center Biostatistics Core, University of Colorado and Children’s Hospital Colorado, Aurora, Colorado, United States of America; 4 Oak Ridge Associated Universities, Oak Ridge, Tennessee, United States of America; 5 OpenPlans, New York, New York, United States of America; 6 Department of Epidemiology, Colorado School of Public Health, University of Colorado Anschutz Medical Campus, Aurora, Colorado, United States of America; 7 Division of Pulmonary Sciences and Critical Care Medicine, School of Medicine, University of Colorado Anschutz Medical Campus, Aurora, Colorado, United States of America; National Institutes of Health, UNITED STATES

## Abstract

The purpose of this study was to assess the validity of a practical diabetes risk score amongst two heterogenous populations, a working population and a non-working population. Study population 1 (n = 2,089) participated in a large-scale screening program offered to retired workers to discover previously undetected/incipient chronic illness. Study population 2 (n = 3,293) was part of a Colorado worksite wellness program health risk assessment. We assessed the relationship between a continuous diabetes risk score at baseline and development of diabetes in the future using logistic regression. Receiver operating curves and sensitivity/specificity of the models were calculated. Across both study populations, we observed that participants with diabetes at follow-up had higher diabetes risk scores at baseline than participants who did not have diabetes at follow-up. On average, the odds ratio of developing diabetes in the future was 1.38 (95% CI: 1.26–1.50, p < 0.0001) for study population 1 and 1.68 (95% CI: 1.45–1.95, p-value < 0.0001) for study population 2. These findings indicate that the diabetes risk score may be generalizable to diverse individuals, and thus potentially a population level diabetes screening tool. Minimally-invasive diabetes risk scores can aid in the identification of sub-populations of individuals at risk for diabetes.

## Introduction

Between 1997 and 2013, on average, the percentage of adults in the United States (US) with diabetes more than doubled from 4.2% to 10.0% [1]. The total cost of diabetes exceeds $245 billion in the US, and approximately 30% of the costs are attributed to permanent disability, work presenteeism, premature mortality, workdays absent, and reduced productivity [2]. While there is evidence that diabetes prevention and treatment programs are successful, many who need the programs are not engaging in them. One reason for this is that many people are unaware that they are at risk for diabetes [3, 4]. Gopalan et al. demonstrated that increasing peoples’ awareness of their risk for diabetes results in greater prevention program participation. One way to increase awareness is through a practical, user-friendly diabetes risk score [4].

A number of minimally invasive diabetes risk scores exist. Commonly incorporated variables are age, gender, smoking, family history of diabetes, and hypertension [5]. Less commonly-included variables are waist circumference, body weight, BMI, ethnic origin, physical activity, and dietary factors such as self-reported consumption of red meat, whole-grain bread, or fruits and vegetables [5, 6]. While researchers have tested many diabetes risk scores and found them to have good predictive value, their results often could not be replicated with different populations [7]. Most scores were developed in populations that are relatively homogenous by geography, age range, or other characteristics [6]. Of particular note is the older age range of most populations in which these measures have been developed. Few of the named diabetes risk scores were developed in populations that included individuals below the age of 35, particularly among U.S.-derived self-report measures [5, 6]. This is significant as type II diabetes is becoming more common in young people in the US. A recent study by insurer Blue Cross Blue Shield found a 4.7% growth in diabetes incidence in individuals ages 18–34 between 2013 and 2015 [8]. This is a population that may not traditionally think of itself at being at risk of developing type II diabetes and has potentially many years of life to reap the benefits of risk factor change. Existing scores also have not been developed explicitly in working populations in the US [6]. Overall, the existing self-report diabetes risk score measures perform well, but have largely not been validated outside of the populations in which they were developed [5].

The purpose of this study is to assess the validity of a practical diabetes risk score. This study is unique in three ways. First, the diabetes risk score is calculated from information that can easily be obtained from self-report health risk assessments, such as those commonly used in worksite wellness programs. Second, this study draws upon two, heterogeneous prospective cohorts of adults in the United States, a working population and a non-working population. As such, this gave us the opportunity to conduct validation tests among two heterogeneous samples thereby maximizing our chances for a generalizable diabetes risk score. Third, we test the diabetes risk score against a self-report measure of diabetes and an objective, clinical measure of diabetes, helping to ensure that the risk score accurately estimates diabetes incidence.

Across both samples, we hypothesized that, among employees without diabetes during their first assessment, participants who have a high diabetes risk score during their first assessment are significantly more likely to have been diagnosed with diabetes by the time of their second assessment than are employees who have a lower diabetes risk score during their first assessment.

## Materials and methods

### Diabetes risk score

Our (type 2) diabetes risk score is based on minimally-invasive self-report information that is weighted to reflect the relative importance of each piece of information. We modeled our risk score after the Finnish Diabetes Risk Score (FINRISK) [9]. Buijsse et al.’s recent review of diabetes risk scores indicated it was the most frequently used and validated score in independent cohorts [7]. It has been tested in samples representative of working-aged populations (<65 years of age) as well as retired populations (>65 years of age) [7]. The FINRISK score is also advantageous as it weights individual risk factors based on how important they are in explaining diabetes risk. However, many of the assessments conducted in workplace and other health promotion surveillance settings do not collect the full suite of information called for in the FINRISK score. We identified this limitation in information available in the health surveillance instruments used in two populations described below, we adapted the FINRISK.

We included the following four of the seven FINRISK variables: age, body mass index (BMI), blood pressure, and random blood glucose. Additionally, we included smoking status, because it has been included in more recently developed risk models [10]. We also included race (white vs. non-white), because ethic origin has been related to diabetes risk [10]. We chose to weight ethnicity higher than smoking as Collins et al.’s [11] review found that ethnicity was more common in risk scores than smoking. We did not include waist circumference, exercise, or history of family members with diabetes, because our surveillance data set did not have information on this. Finally, we did not include vegetable, fruit, or berry consumption because in preliminary analyses with our samples, it did not demonstrate a significant bi-variate relationship with diabetes with study population 2.

We evaluated the diabetes risk score as a continuous measure of disease risk. The risk score ranged from 0 to 19 from low to high risk, respectively. Within our diabetes risk score, blood glucose held the greatest weight followed by age, BMI, race, smoking status, and blood pressure. The list of questions for each risk factor for both samples is presented in Table 1.

**Table 1:** Description of variables used in each study population to calculate the diabetes risk score and estimate diabetes at follow-up.

Study population 1			Study population 2		
Diabetes risk score variable	Point contribution to risk score	Response option	Diabetes risk score variable	Point contribution to risk score	Response option
**Age**		“Age” [Enter age]	**Age**		Same as study population 1
	0	<45		0	
	2	45–53		2	
	3	55–64		3	
	4	65+		4	
**Race**		“Select race/ethnicity”	**Race**		Same as study population 1
	0	1 () White/Caucasian		0	
	3	2 () African-American		3	
	3	3 () Hispanic/Latino		3	
	3	4 () Asian		3	
	3	5 () Native American		3	
	3	6 () Other		3	
	3	7 () East Indian		3	
	3	8 () Middle Eastern		3	
**Current smoker**		Ever Smoker:	**Current smoker**		“Smoking status”
				0	1 () never smoked
	0	No (0)		0	2 () quit smoking
	2	Yes (2)		2	3 () presently smoke
**Blood pressure**		High Blood Pressure (Systolic/Diastolic ≥ 140/90):	**Blood pressure**		“Mark medications taken regularly: Blood pressure lowering”
	2	Yes (2)		2	1 () blood pressure lowering
	0	No (0)		0	2 () no
**BMI**		Calculated from “Height” and “Weight”	**BMI**		Same as study population 1
		[Enter height]			
		[Enter weight]			
	0	<25		0	
	1	25–30		1	
	3	>30		3	
**Blood glucose**		Elevated Clinical Blood Glucose Value: Fasting (100–125) or Random (139–199):	**Blood glucose**		“Mark your values: Glucose (fasting) (normal<100, high>126)”
				0	1 () normal
				5	2 () moderately elevated (between normal and high)
	5	Yes (5)		5	3 () high
	0	No (0)		0	4 () don’t know
**Diabetes at 3-year follow-up–**Clinical interpretation of blood glucose values^2,13^		Fasting glucose value > = 126 mg/dL	**Diabetes at 1 year follow-up–**Self-report diabetes		“Do you have diabetes?”
		or			No diabetes = “no I don’t have this condition”
		Non-fasting (random) value > = 200			Diabetes = “yes but I never received professional treatment” or “yes I previously received (but don’t currently receive) professional treatment” or “yes and I currently receive professional treatment”

It is important to note that each variable included in the risk score, except for blood glucose, came from self-reported information across both samples. Blood glucose was measured as part of a Chem-22 panel at the time of survey administration in study population 1. Blood glucose level was based on the self-reported response to a survey question in study population 2.

### Study population 1

The first study population came from the National Supplemental Screening Program (NSSP). The NSSP is part of the Department of Energy (DOE) Former Worker Program, established by the National Defense Authorization Act to assist workers in determining whether they have health issues related to their past work with at DOE facilities. The NSSP takes a Total Worker Health^®^ (TWH) approach to former DOE worker health, both screening for potential work-related medical conditions related to past DOE site employment and providing screening for non-occupational health conditions [12]. From October 2005 to December 2015, the NSSP has provided initial medical examinations to more than 15,000 former DOE workers living in 47 US states. The study population represents former Department of Energy Workers from a variety of occupations, with an average age of 64.

As part of the screening program, former workers complete the NSSP Health and Exposure History questionnaire, a clinical exam by a licensed practitioner, and laboratory testing which includes a complete metabolic panel [12]. The NSSP interviewer asks health history questions related to health behavior such as weight loss and smoking as well as current medical diagnoses. As part of the medical screening program, venipuncture was performed, and non-fasting serum glucose was measured as part of the Chem-22 panel. In this sample, diabetes at follow-up was measured via clinical interpretation of blood glucose values (see Table 1). While individuals are eligible for rescreening after 3-years the actual time between their initial exam and follow-up exam may have been longer. The median for this cohort was 5 years (interquartile range 3–7 years).

To be included in the present study, former workers must have completed at least two screenings (n = 2651), not have clinical glucose values indicating diabetes at the time of first assessment (n = 2534), and not have missing data on any of the self-reported variables that contribute to the risk score. The final number of workers included in this sample was 2,089. The investigators neither recruited participants nor collected new data for this study. All data were de-identified by NSSP staff before being sent to the investigators. Thus, approval from the Department of Energy Central Institutional Review Board was obtained with exempt status, with additional informed consent not required.

### Study population 2

The second sample came from the Pinnacol Assurance Health Risk Management Study [13, 14]. The study population represents working adults, ages 18–65, in Colorado, from a variety of industries and occupations. Workers in the HRM study were part of an effort to evaluate a worksite wellness program administered by a local workers’ compensation firm from May 2010 to December 2014.

As part of the study, workers completed a Wellsource^®^ health risk assessment (HRA) (Tualatin, OR) via an online, self-administered English and Spanish survey. The HRA included a variety of questions related to demographics, health behaviors, and mental health. It was supplemented with the WHO Health and Work Performance Questionnaire, that included questions about chronic health conditions and productivity [15]. In this sample, diabetes at follow-up was measured via a self-report survey question (see Table 1).

A total of 16,926 employees participated in the survey. However, to be included in the present study, workers must have completed at least two health risk assessments approximately one year apart (n = 5766), not indicate that they had diabetes during their first health risk assessment by responding “no I don’t have this condition” to the “Do you have diabetes?” question (n = 5563). Additionally, patients could not have missing data on any of the self-reported variables that contribute to the risk score, which resulted in 3,293 workers included in this sample. The Colorado Multiple Institutional Review Board deemed this study to be non-human subjects research.

### Analysis

We used logistic regression to assess the ability of the continuous diabetes risk score to estimate diabetes onset at follow-up, which could occur as early as 3-years after their initial exam in study population 1 and one year later in study population 2. Because we were unable to split the dataset into a test and training set and use cross validation due to the small number of cases of diabetes in each sample, we used bootstrap resampling for modeling training and validation. Consequently, estimates may overestimate model performance when applied to an external dataset. The linearity assumption for risk score on the logit scale was assessed and appeared to be valid for both samples (results not shown). The models were trained using bootstrap resampling (1000 x) and the final model was selected based on the average model from the resampling. Predictive value was evaluated through receiver operating curves (ROC) and calculation of sensitivity and specificity of the model for each sample. Bootstrap resampling (1000 x) was used to evaluate variation in the estimates. All analyses were performed using R version 3.2.4.

## Results and discussion

### Study population description

As expected, we observed some variability in study population demographics between study population 1 (former workers, national program) and 2 (currently employed workers, Colorado program). Compared to study population 2, the majority of participants in study population 1 were older, male, and worked in blue collar occupations, such as the craft/operator/laborer category (see Table 2). In population 2, 0.69% (n = 23) of the sample reported diabetes at follow-up one-year later whereas 2.70% (n = 57) of study population 1 had clinical blood glucose values indicating diabetes at follow-up, which could have occurred as early as 3-years after their initial exam.

**Table 2:** Baseline demographic characteristics of study population 1 (US former DOE workers, 2005–2015) and study population 2 (Colorado workers from multiple businesses, 2010–2014).

Characteristic at baseline	Study Populations	
	Study population 1 (n = 2,089)	Study population 2 (n = 3,293)
	No. (%)	No. (%)
Age		
< 45	89 (4.26)	1,635 (49.65)
45–54	364 (17.42)	967 (29.37)
55–64	580 (27.76)	613 (18.62)
65+	1056 (50.55)	78 (2.37)
Gender		
Female	493 (23.6)	2,200 (66.81)
Male	1596 (76.4)	1,093 (33.19)
Race		
White	1786 (85.5)	2,935 (89.13)
Non-White	303 (14.5)	359 (10.87)
Current Smoker		
Yes	147 (7.04)	233 (7.08)
No	1942 (92.96)	3,060 (92.92)
Occupation		
Management	48 (2.30)	504 (15.31)
Professional	803 (38.44)	1,468 (44.58)
Administrative/Technical Support	430 (20.58)	610 (18.52)
Service	264 (12.64)	358 (10.87)
Craft/Operator/Laborer	508 (24.32)	239 (7.26)
Unknown	26 (1.72)	114 (3.46)
Region		
Midwest	673 (32.22)	0 (0.00)
Northeast	70 (3.35)	0 (0.00)
South	661 (31.64)	0 (0.00)
West	685 (32.79)	100 (100.00)

The only difference in baseline characteristics between study populations was in gender whereby there were more male diabetics at follow-up in study population 1 and more females in study population 2. Across both study populations, a greater proportion of diabetic participants were older, smokers, hypertensive, obese, non-white, and had elevated blood glucose.

Ultimately, across both study populations, we observed that participants who were found to have diabetes at follow-up had higher diabetes risk scores at baseline than did participants who did not have diabetes at follow-up (Table 3). In study population 1, diabetics risk score at baseline was 8.46 (SD = 3.42), but non-diabetics was 5.83 (SD = 2.49). In study population 2, diabetes risk score at baseline was 6.78 (SD = 2.88) and non-diabetics was 2.93 (SD = 2.40).

**Table 3:** Demographic characteristics and risk scores by diabetes status at re-screening for study population 1 (US former DOE workers, 2005–2015) and study population 2 (Colorado workers from multiple businesses, 2010–2014).

Characteristic at baseline	Diabetes status at re-screening			
	Study population 1		Study population 2	
	Non-Diabetic (n = 2032)	Diabetic (n = 57)	Non-Diabetic (n = 3270)	Diabetic (n = 23)
	No. (%) or Mean (SD^a^)	No. (%) or Mean (SD)	No. (%) or Mean (SD)	No. (%) or Mean (SD)
Age				
< 45	86 (4.2)	3 (5.3)	1631 (49.9)	4 (17.4)
45–54	357 (17.6)	7 (12.3)	958 (29.3)	9 (39.1)
55–64	563 (27.7)	17 (29.8)	606 (18.5)	7 (30.4)
65+	1026 (50.5)	30 (52.6)	75 (2.3)	3 (13.0)
Gender				
Female	485 (23.9)	8 (14.0)	2,183 (66.76)	17 (73.91)
Male	1547 (76.1)	49 (86.0)	1,087 (33.24)	6 (26.09)
Current Smoker				
No	1890 (93.0)	52 (91.2)	3042 (93.0)	18 (78.3)
Yes	142 (7.0)	5 (8.8)	228 (7.0)	5 (21.7)
Blood Pressure				
Normal	1918 (94.4)	49 (86.0)	2849 (87.1)	11 (47.8)
High	114 (5.6)	8 (14.0)	421 (12.9)	12 (52.2)
BMI				
< 25	449 (22.1)	4 (7.0)	1493 (45.7)	3 (13.0)
25–30	890 (43.8)	11 (19.3)	1120 (34.3)	6 (26.1)
> 30	693 (34.1)	42 (73.7)	657 (20.1)	14 (60.9)
Blood Glucose				
Normal	1875 (92.3)	38 (66.7)	3249 (99.4)	21 (91.3)
Elevated	157 (7.7)	19 (33.3)	21 (0.6)	2 (8.7)
Race				
White	1742 (85.7)	44 (77.2)	2917 (89.2)	18 (78.3)
Non-White	290 (14.3)	13 (22.8)	353 (10.8)	5 (21.7)
Continuous diabetes Risk Score Mean (SD)	5.73 (2.49)	8.46 (3.43)	2.93 (2.4)	6.87 (2.88)

^a^Standard deviation

### Validation results

Table 4 presents the logistic regression results for the diabetes risk scores estimating future incidence of diabetes.

**Table 4:** Logistic regression results for continuous diabetes risk scores estimating future incidence of diabetes for study population 1 (US former DOE workers, 2005–2015) and study population 2 (Colorado workers from multiple businesses, 2010–2014).

Diabetes Risk Score	Odds Ratio	Lower 95% CI^a^	Upper 95% CI	P-Value
*Study Population 1*	1.38	1.26	1.50	< 0.0001
*Study Population 2*	1.68	1.45	1.95	< 0.0001

^a^CI = Confidence interval.

#### Study population 1

In study population 1, we observed a significant relationship between the diabetes risk score and the development of diabetes upon rescreening. On average, the odds ratio of developing diabetes in the future was 1.38 (95% CI: 1.26–1.50, p < 0.0001) for every unit increase in the diabetes risk score. We evaluated the final model’s ability to correctly identify one as having future diabetes based on their risk score using bootstrap resamples (1000x) from the full dataset, which yielded an average area under the receiver operating characteristic curve (AUC) of 74.5% (95% CI: 74.3–74.7). A specificity of 66.1% and a sensitivity of 77.2% was obtained using Youden’s index (i.e., max(sensitivity + specificity) to determine the a cutoff of 0.024. Thus, while 77.2% will be correctly identified as diabetic based on their risk score, there is a high false positive rate (34%) of incorrectly identifying individuals as diabetic based on their risk score. The number of true positives was 44, false positives 688, true negatives 1344, and false negatives 13. This resulted in a positive predictive value (PPV) of 6% and a negative predictive value (NPV) of 99%. Thus, of those that test positive for developing diabetes at next rescreening exam, only 6% will actually develop diabetes. Yet, 99% of those that test negative will not go on to develop diabetes within the next three years. However, the low prevalence of future diabetes in our study cohort (2.7%) is a major influence on the PPV and NPV seen here. For example, if we took a naive approach and labeled everyone as negative, the NPV would be 97.3%. Lastly, the positive likelihood ratio was 2.28. Therefore, a positive test result is 2.28 times more likely for individuals with diabetes than those without.

#### Study population 2

Table 4 also presents the logistic regression results for the association of the diabetes risk score with incidence of diabetes for study population 2. We observed a significant relationship between the diabetes risk score and the development of diabetes in the future in study population 2. On average, the odds ratio of developing diabetes in the future was 1.68 times higher for every unit increase in the diabetes risk score (95% CI: 1.45–1.95), p-value < 0.0001). The AUC was 85.0% (95% CI: 84.7–85.3), and using Youden’s index to determine a cutoff value of 0.006 resulted in a specificity of 74.9% and a sensitivity of 87.0%. Thus, while 87.0% will be correctly identified as diabetic based on their risk score, there is a high false positive rate (25%) of incorrectly identifying individuals as diabetic based on their risk score. The, number of true positives was 20, false positives 822, true negatives 2448, and false negatives 3. This resulted in a positive predictive value (PPV) of 2.4% and a negative predictive value (NPV) of 99.9%. Thus, of those that test positive for developing diabetes at next rescreening exam only 2.4% will actually develop diabetes. Yet, 99.9% of those that test negative will not go on to develop diabetes within the next year. Lastly, the positive likelihood ratio was 3.5.

## Discussion

Although many studies have already developed diabetes risk scores for the purpose of predicting disease onset, researchers have argued that in order to move this field forward and have value in diabetes prevention, the scores should undergo predictive validity testing [6]. Our study addresses this suggestion by adapting a previously developed risk score and validating it in two heterogenous populations at multiple follow-up time points. We draw two major conclusions. First, our diabetes risk score is positively associated with the future development of diabetes measured either via self-report survey or with inclusion of clinical laboratory values for blood glucose. Second, our diabetes risk score may be generalizable to diverse individuals, and thus potentially a population level diabetes screening tool. The next step in this research should be to understand how the score can be used in practice to promote disease awareness and health behavior change.

Many studies have noted the value of minimally-invasive diabetes risk scores in clinical care settings, but only one has noted the potential value in work settings as a tool for health promotion [16, 17]. We tested the score in two “non-clinical” settings in which the ability to assess risk for diabetes is important, i.e. as part of worksite wellness program health risk assessments, and in large scale screening programs offered to retired workers to discover previously undetected or incipient chronic illness. Cited reasons for value of minimally-invasive health tests in clinical settings include ease of use, increased comfort, decreased risk compared to more invasive measures, and cost effectiveness [5, 18]. Working populations spend approximately one-third of their lives at work, and as such the work environment can be an opportune place to screen for diabetes risk. Indeed, workplace screenings could reach people who may not otherwise regularly see their primary care physician. Furthermore, many employers are moving towards promoting overall health of their workforce as they seek to increase workforce engagement and productivity, reduce healthcare costs related to chronic health conditions as well as workers’ compensation claims and costs [19, 20].

The Kaiser Family Foundation 2014 Health Benefits Survey found 1/3 of employers offered health risk assessment to their employees, including 36% of large firms with greater than 200 employees [21]. These assessments are commonly used as an intervention to help employees understand their health risks and sometimes as the basis for health coaching. When used as a risk assessment tool, a diabetes risk score derived from the assessment could provide a large number of Americans access to basic information which could be used to help them better understand their risk for diabetes without undergoing any further testing. It can also be used by employers to guide worksite wellness program design and allocation of resources for diabetes prevention. Given the low-cost and ease of use, a diabetes risk score from an HRA can be an accessible health promotion intervention for businesses of all sizes.

Few studies have tested diabetes risk scores in an intervention setting to understand if and how individuals change their modifiable diabetes risk factors in response to a high-risk score [5, 6]. Given the prevalence of HRAs among employers in the US and the rising prevalence of diabetes in working populations, the workplace is an ideal setting in which to test the diabetes risk score as an intervention to prevent the development of diabetes. Workers could use their diabetes score from the health risk assessment to understand and address their diabetes risk, such as participation in a diabetes prevention program, education on exercise and nutrition, and closer medical monitoring [3]. Additionally, the diabetes risk score could help wellness health coaches provide preventative advice when reviewing a client’s HRA data, making HRA data more useful. Furthermore, researchers should test whether the use of this score over time is helpful as a metric in evaluation of the return on investment for using the minimially-invasive risk score versus more invasive laboratory test. Results of these studies can provide evidence needed for business investment in diabetes prevention programs.

A strength of this study is the generalizability of the findings, reaching a large, nationally representative sample of retired workers as well as a large population of currently employed Coloradans from across the state. It also suggests that a very short survey, even in the absence of blood glucose determination, has value as part of a health risk assessment.

We were limited by the low number of individuals who went on to develop diabetes at follow-up. This small sample may have contributed to the less than optimal specificity we observed as well as the unstable estimates at high and very high risk for developing diabetes. Additionally, the short follow-up time for study population two may have hindered our ability to detect the onset of diabetes in this population. Relatedly, it is interesting that study population 2, a working population, had a higher odds of developing diabetes than study population 1, a retired population. We believe this may be due either retirees being less likely to attend a medical screening or a survivor effect. Finally, due to strict inclusion criteria, our study reflects only a small proportion of the two study populations. While the baseline demographics for the populations are similar to the overall populations [12, 22], the results should still be interpreted with caution.

Another limitation is the small sample size did not allow for splitting the data into separate training and test sets and model performance presented here may be overestimated. These results could be validated in the future by assessing model prediction on an external dataset. An inherent limitation of HRA data is the reliance on self-report data. It would be worth evaluating the diabetes risk score against HbA1c to obtain a more accurate estimate of the relationship between the risk score and future incidence of diabetes. It may be especially important to include an objective measure of blood pressure as the relationship between blood pressure and diabetes has a j-curve. Unfortunately, we did not have access to the necessary objective blood pressure values to evaluate wither this curve affects the risk score.

Diabetes risk scores can aid in the identification of sub-populations of individuals at risk for diabetes. They can be easily used in a variety of settings such as clinical care, workplace wellness programs, community health centers, among others. In public health practice, the aggregate results of a workforce’s diabetes risk score could be used to determine the need for employers to invest in diabetes prevention programs, based on the proportion of workers at risk of diabetes.
